# Functional and cardioprotective effects of simultaneous and individual activation of protein kinase A and Epac

**DOI:** 10.1111/bph.13709

**Published:** 2017-02-14

**Authors:** Igor Khaliulin, Mark Bond, Andrew F James, Zara Dyar, Raheleh Amini, Jason L Johnson, M‐Saadeh Suleiman

**Affiliations:** ^1^ School of Clinical Sciences and Bristol CardiovascularUniversity of BristolBristolUK

## Abstract

**Background and Purpose:**

Myocardial cAMP elevation confers cardioprotection against ischaemia/reperfusion (I/R) injury. cAMP activates two independent signalling pathways, PKA and Epac. This study investigated the cardiac effects of activating PKA and/or Epac and their involvement in cardioprotection against I/R.

**Experimental Approach:**

Hearts from male rats were used either for determination of PKA and PKC activation or perfused in the Langendorff mode for either cardiomyocyte isolation or used to monitor functional activity at basal levels and after 30 min global ischaemia and 2 h reperfusion. Functional recovery and myocardial injury during reperfusion (LDH release and infarct size) were evaluated. Activation of PKA and/or Epac in perfused hearts was induced using cell permeable cAMP analogues in the presence or absence of inhibitors of PKA, Epac and PKC. H9C2 cells and cardiomyocytes were used to assess activation of Epac and effect on Ca^2+^ transients.

**Key Results:**

Selective activation of either PKA or Epac was found to trigger a positive inotropic effect, which was considerably enhanced when both pathways were simultaneously activated. Only combined activation of PKA and Epac induced marked cardioprotection against I/R injury. This was accompanied by PKCε activation and repressed by inhibitors of PKA, Epac or PKC.

**Conclusion and Implications:**

Simultaneous activation of both PKA and Epac induces an additive inotropic effect and confers optimal and marked cardioprotection against I/R injury. The latter effect is mediated by PKCε activation. This work has introduced a new therapeutic approach and targets to protect the heart against cardiac insults.

**Abbreviations**
CaMKII: Ca^2+^/calmodulin‐dependent protein kinase II
CPT: 8‐(4‐chlorophenylthio)‐2′‐O‐methyladenosine‐3′,5′‐cyclic monophosphate, acetoxymethyl ester (8‐pCPT‐2′‐O‐Me‐cAMP‐AM)
ESI‐09: 3‐[5‐(tert.‐butyl)isoxazol‐3‐yl]‐2‐[2‐(3‐chlorophenyl)hydrazono]‐3‐oxopropanenitrile
HR: heart rate
I/R: ischaemia/reperfusion
IP: ischaemic preconditioning
KH: Krebs–Henseleit buffer
LVDP: left ventricular developed pressure
MPTP: mitochondria permeability transition pore
PKI: PKA inhibitor 14–22 amide
RPP: rate‐pressure product
RyR2: ryanodine receptor
6‐Bnz: N6‐benzoyladenosine‐3′,5′‐cyclic monophosphate, acetoxymethyl ester (6‐Bnz‐cAMP‐AM)
8‐Br: 8‐bromoadenosine‐3′,5′‐cyclic monophosphate, acetoxymethyl ester (8‐Br‐cAMP‐AM)
+dP/dt: time derivative of pressure measured during contraction
‐dP/dt: time derivative of pressure measured during relaxation

## Tables of Links

**Table:** 

**TARGETS**
**GPCRs** ^*a*^
β‐adrenoceptors
**Enzymes** ^*b*^
EPACs
PKA
PKCε
PKCδ

**LIGANDS**
6‐Bnz, 6‐Bnz‐cAMP‐AM, N^6^‐benzyl cyclic AMP
CPT, 8‐pCPT‐2’‐O‐Me‐cAMP‐AM
cAMP
Chelerythrine
Isoprenaline
H‐89

These Tables list key protein targets and ligands in this article which are hyperlinked to corresponding entries in http://www.guidetopharmacology.org, the common portal for data from the IUPHAR/BPS Guide to PHARMACOLOGY (Southan *et al*., 2016), and are permanently archived in the Concise Guide to PHARMACOLOGY 2015/16 (^*a,b*^Alexander *et al*., 2015a,b).

## Introduction

Cardiac ischaemia/reperfusion (I/R) injury can occur during coronary angioplasty, cardiac surgery and heart transplantation (Depre and Taegtmeyer, 2000) contributing to morbidity and mortality (Eltzschig and Eckle, 2011). This injury is mediated by the opening of the mitochondria permeability transition pore (MPTP), which can be triggered by Ca^2+^ overload (Halestrap *et al.,* 2004) and ROS (Honda *et al.,* 2005; Halestrap and Pasdois, 2009). We and others have provided evidence showing that the cAMP signal transduction pathways are involved in mediating a number of cardioprotective effects (Lochner *et al.,* 1999; Khaliulin *et al.,* 2010; Khaliulin *et al.,* 2014). Our work has also identified PKC as a critical component of cAMP/PKA‐induced cardioprotection (Khaliulin *et al.,* 2007; Khaliulin *et al.,* 2010). A major problem associated with targeting the cAMP/PKA pathway is the fact that this treatment has relied on stimulation of β‐adrenoceptors, which may be impaired in chronic heart failure due to hyperstimulation of β‐adrenoceptors, mediated via the sympathetic nervous system (Cannavo *et al.,* 2013; Lymperopoulos *et al.,* 2013). Therefore, if the protective cAMP‐related signalling mechanisms can be activated without stimulation of β‐adrenoceptors, this would give a significant therapeutic advantage. Although most of the biological effects of cAMP on the heart have been assigned to PKA (Bers, 2007), cAMP also activates Epac (a guanine nucleotide exchange protein directly activated by cAMP) (de Rooij *et al.,* 1998). The cAMP/Epac pathway is independent of and parallel to the cAMP/PKA signalling pathway (Pereira *et al.,* 2012; Okumura *et al.,* 2014). Therefore, availability of cell‐permeable cAMP analogues allowing selective activation of either PKA or Epac represents a valuable tool to identify the involvement and the contribution of these cAMP sensors in cardioprotection (Dudley *et al.,* 2014). cAMP analogues have been successfully used in work involving vascular smooth muscle cell proliferation where both PKA and Epac were found to act synergistically at inhibiting proliferation (Hewer *et al.,* 2011; Kimura *et al.,* 2014). In this study, we investigated the cardioprotective efficacy of cAMP/PKA and cAMP/Epac signalling pathways using cell permeable cAMP analogues that are selective activators of either PKA or Epac or both and assessed the involvement of PKC in the cAMP‐induced cardioprotection.

## Methods

### Animals

All animal care and experimental procedures conformed to the Animals (Scientific Procedures) Act, 1986 of the UK Parliament, Directive 2010/63/EU of the European Parliament and the Guide for the Care and Use of Laboratory Animals published by the US National Institutes of Health (NIH Publication No. 85–23, revised 1996). Ethical approval was granted by the Animal Welfare and Ethics Review Board of the University of Bristol, UK. Animal studies are reported in compliance with the ARRIVE guidelines (Kilkenny *et al.*, 2010; McGrath and Lilley, 2015).

The experiments were performed using 2‐month‐old male Wistar rats (250–260 g) supplied by Charles River Laboratories (Oxford, UK). Prior to experiments, the animals were housed at the University of Bristol Animal Services Unit for 2–4 days at 23 ± 1°C, with a relative humidity of 60–70% and a light/dark cycle of 12 h with free access to water and standard rat chow. The rats were kept in standard cages, two to three animals per cage, with aspen bedding. The rats were killed by stunning and cervical dislocation and used for experiments on isolated heart and cardiomyocytes. Freshly isolated rat cardiomyocytes and the H9C2 cell line were used for validation and optimization of PKA and Epac activation induced by the cell‐permeable cAMP analogues: 8‐bromoadenosine‐3′,5′‐cyclic monophosphate, acetoxymethyl ester (8‐Br); N^6^‐benzoyladenosine‐3′,5′‐cyclic monophosphate, acetoxymethyl ester (6‐Bnz) and 8‐(4‐chlorophenylthio)‐2′‐O‐methyladenosine‐3′,5′‐cyclic monophosphate, acetoxymethyl ester (CPT).

### H9C2 cell culture

Rat heart cardiomyoblasts H9C2 were incubated in plates (150 000 cells·mL^−1^) at 37°C in DMEM (Invitrogen Gibco, Paisley, UK) with L‐glutamine (1%), penicillin (100 U·mL^−1^) and streptomycin (100 U·mL^−1^) for 7 h with either 5 μM 8‐Br, 5 μM 8‐Br + 1 μM ESI‐09 or 10 μM CPT. RNA of these cells was extracted, transcribed to cDNA, and expression of the gene c‐fos, used as an indicator of Epac activation (Glas *et al.,* 2016), was assessed by qRT‐PCR. The average c‐fos expression of three experiments was calculated for each group of cells. The gene expression was calculated as fold change relative to the control. The level of gene expression in the control groups was set as 1.

H9C2 cells were selected because they are rat heart cardiomyoblasts and therefore represent a rat cardiomyocyte precursor cell‐type. Moreover, H9C2 cells in culture are very robust and allow experiments of prolonged duration to be conducted. In particular, this allowed us to perform the determination of c‐fos expression in cells treated with 8‐Br, CPT and the Epac inhibitor, ESI‐09.

### Assessment of PKA and PKC activation in isolated rat heart

PKA activation was assessed by measuring phosphorylation of VASP with phospho‐VASP (Ser^157^) antibody (rabbit; from Cell Signalling Technology, Inc., NEB, Hitchin, UK; diluted 1:1000) by Western blots and using an elisa‐based PKA kinase activity assay.

For detection of VASP phosphorylation, proteins were extracted from powdered frozen ventricular cardiac tissue from rat hearts that were perfused in the Langendorff mode with different drugs according to the protocols described below. The protein extraction buffer contained (mM) the following: 300 sucrose, 10 Tris–HCl, 1 EGTA, 2 sodium pyrophosphate, 2 sodium fluoride, 2 β‐glycerophosphate, complete protease inhibitor cocktail (Roche Diagnostics, Burgess Hill, UK) and phosphatase inhibitor cocktail 3 (Sigma, Gillingham, UK). Separation of the cytosol and membrane fractions of the heart samples was carried out as described earlier (Clarke *et al.,* 2008). In brief, cell debris were removed from protein extraction buffer by centrifugation at 2000 × *g* for 1.5 min. The supernatant was centrifuged at 200 000 × *g* for 1 h, and the resulting supernatant was taken as the cytosolic fraction. The pellet (membrane fraction) was resuspended in the protein extraction buffer. After diluting the samples (1:1 v·v^−1^) with the SDS sample buffer containing at final concentration: 50 mM Tris–HCl, 2 mM EDTA, 12% glycerol and 10% SDS, protein concentration was adjusted to 2 mg·mL^−1^ using BCA Protein Assay (Thermo Fisher Scientific, Loughborough, UK). Then, 2‐mercaptoethanol and bromophenol blue were added at final concentrations of 5% and 0.01%, accordingly. All the procedures of the protein separation were carried out at 4°C. Lysate from forskolin‐stimulated rat smooth muscle cells was used as a positive control for evaluation of PKA activation by VASP phosphorylation. Protein loading was assessed with anti‐GAPDH antibody (Cell Signalling Technology, Inc., NEB, Hitchin, UK; diluted 1:8000).

An additional method was also employed to determine PKA activation using an elisa‐based PKA kinase activity assay kit (ab139435, Abcam, Cambridge, UK). This kit was used for measuring the PKA activity in the lysates of frozen ventricular cardiac tissue. The frozen heart powders were mixed with the lysis buffer containing (mM) the following: 20 MOPS, 50 β‐glycerolphosphate, 5 EGTA, 2 EDTA, 1% NP40, complete protease inhibitor cocktail (Roche Diagnostics, West Sussex, UK) and phosphatase inhibitor cocktail 3 (Sigma, Gillingham, UK). The samples were centrifuged at 2000 × *g* for 5 min at 4°C to remove the cell debris. Protein concentration was adjusted to 4 mg·mL^−1^ using BCA protein assay. About 2 μg of protein of each sample were assayed according to the manufacturer's instructions but without the addition of 0.5 mM of ATP to the heart samples (Supplementary Information Fig. S3).

Activation of PKCε and PKCδ was assessed by translocation of these PKC isoforms from the cytosol to membrane fraction (Mochly‐Rosen *et al.,* 1990). Samples (20 μg per well) containing the cytosol and membrane fractions were separated by 10% SDS‐PAGE and subjected to Western blotting with anti‐PKCε; and anti‐PKCδ primary antibody (rabbit; from Cell Signalling Technology, Inc., NEB, Hitchin, UK; diluted 1:1000). Each blot contained samples of both cytosol and membrane fractions from the hearts of control and 8‐Br‐treated hearts to allow direct comparison (Figure 3A–B). Membrane‐to‐cytosol fraction ratio of the optical density was used to evaluate activation of PKCε and PKCδ. Equal protein loading was confirmed by staining of the PVDF membranes with Ponceau‐S stain for 2 min at room temperature followed by wash with tap water (Supplementary Information Fig. S4).

All blots were scanned with an HP scanner, and quantification of band intensity was performed using AlphaEase v5.5 software followed by background subtraction.

### Cardiomyocyte isolation and superfusion

The aim of these experiments was to establish whether the strong inotropic response produced by 8‐Br, which activates both PKA and Epac, is mediated by an increase in intracellular Ca^2+^ transients that is comparable with isoprenaline, which we have previously found to induce a strong cardioprotective effect (Khaliulin *et al.,* 2014). We used an established model of monitoring Ca^2+^ transient in isolated adult rat cardiomyocytes. Rat cardiomyocytes were isolated using collagenase Type I (Worthington Biochemical Corporation, Lakewood, New Jersey, USA) and protease as described previously (Williams *et al.,* 2001). Cells were loaded with Fura‐2‐AM (5 μM; Biotium, Cambridge, UK), and Ca^2+^ transients were recorded at the excitation/emission of 415/510 nm using a photomultiplier as described elsewhere (Littlejohns *et al.,* 2014). Briefly, cardiomyocytes were superfused at a rate of 0.4 mL·min^−1^ (32–33°C) with HEPES buffer solution consisting of (mM) as follows: 137 NaCl, 5 KCl, 1.2 MgSO_4_·7H_2_O, 1.2 NaH_2_PO4·2H_2_O, 20 HEPES, 15 D‐glucose anhydrous and 2 CaCl_2_ (pH 7.4). The stimulation voltage was set at just above the threshold required for the cell to beat. The photomultiplier was connected to Felix 32 Analysis v1.2 software (Photon Technology International, USA). After a 20 min equilibration period, cells were superfused with a HEPES buffer containing either 10 nM isoprenaline or 5 μM 8‐Br for 5 min followed by 5 min washout.

### Langendorff perfusion of rat isolated hearts

Rats were killed as described above, hearts (~0.75 g) were rapidly removed into ice‐cold Krebs–Henseleit buffer (KH) containing (mM) the following: 118 NaCl, 25 NaHCO_3_, 4.8 KCl, 1.2 KH_2_PO_4_, 1.2 MgSO_4_, 11 glucose and 1.2 CaCl_2_, gassed with 95% O_2_–5% CO_2_ at 37°C (pH 7.4) and perfused in Langendorff mode as described previously (Khaliulin *et al.,* 2011). All hearts were allowed an equilibration period of at least 25 min before any additional treatment was given. Data acquisition and analysis used a PowerLab System (ADInstruments Ltd, Oxford, UK). Left ventricular developed pressure (LVDP) was calculated as the difference between left ventricular systolic pressure and left ventricular end diastolic pressure. Work index (i.e. rate‐pressure product, RPP) was calculated as the product of LVDP and heart rate (HR). Time derivatives of pressure measured during contraction (+dP/dt) and relaxation (−dP/dt) were computed using the software Chart 5 (ADInstruments Ltd, Oxford, UK). Pre‐ischaemic values of the haemodynamic parameters were measured at the end of the equilibration period prior to any pre‐ischaemic intervention. As the hearts in our experiments beat spontaneously, haemodynamic function is reflected in both LVDP and HR. For this reason, RPP was considered as the main parameter characterizing haemodynamic function. If RPP was less than 15 000 mmHg·beat·min^−1^ at the end of the pre‐ischaemic equilibration period, the heart was excluded from the experiment. The absolute values of the haemodynamic indices are shown in the Tables 1 and 2. For analysis of RPP response to heart perfusion with the cAMP analogues and inhibitors, the data were presented as percentage of pretreatment value (Figure 2). This normalization was performed for clarity and in order to avoid unwanted variations of the absolute values.

**Table 1:** Effects of 8‐Br with or without ESI‐09 (inhibitor of Epac), H‐89 or PKI (inhibitors of PKA) and chelerythrine (Chel; inhibitor of PKC) on haemodynamic function recovery during reperfusion

Parameters	LVDP (mmHg)	HR (beat·min^−1^)	RPP (mmHg·beat·min^−1^)·10^3^	+dP·dt^−1^ (mmHg·s^−1^)·10^2^	−dP·dt^−1^(mmHg·s^−1^)·10^2^
Pre‐ischaemic equilibration (*n* = 58)	64.8 ± 2.4	294.3 ± 5.4	19.1 ± 0.8	28.4 ± 1.1	−20.3 ± 1.0
Reperfusion					
Experimental Series 1					
Control (*n* = 7)	19.0 ± 4.0***	289.0 ± 21.8	5.5 ± 1.0***	9.9 ± 1.1***	−9.6 ± 1.0***
8−Br (5 μM, *n* = 7)	64.5 ± 7.6*	262.7 ± 10.5	17.3 ± 2.6*	23.2 ± 3.1*	−16.0 ± 2.3*
8−Br + ESI−09 (*n* = 7)	23.3 ± 8.0**	231.1 ± 13.0*	5.4 ± 1.9**	8.7 ± 2.2**	−7.9 ± 1.6**
8−Br + H−89 (*n* = 6)	26.1 ± 4.9** ***	202.8 ± 25.5*	5.0 ± 1.3**	10.0 ± 1.6**	−8.5 ± 1.2**
ESI−09 (*n* = 7)	19.2 ± 4.0** ***	264 ± 10.8	5.1 ± 1.1** ***	8.7 ± 1.4** ***	−8.8 ± 1.4** ***
Experimental series 2					
Control (*n* = 7)	32.5 ± 6.7***	300.4 ± 5.5	8.6 ± 1.3***	13.7 ± 2.6***	−12.2 ± 2.4***
8−Br (10 μM, *n* = 7)	97.4 ± 9.1* ***	245.4 ± 11.6*	23.7 ± 2.7*	34.2 ± 3.8*	−23.9 ± 2.3*
8−Br + Chel (*n* = 5)	102.9 ± 7.4*	228.2 ± 14.9*	23.4 ± 2.1*	39.6 ± 3.2*	−26.9 ± 2.4*
8−Br + PKI (*n* = 5)	57.8 ± 7.1*	294.0 ± 11.4	17.1 ± 2.4*	24.4 ± 3.4*	−20.2 ± 2.4*
PKI (*n* = 5)	37.2 ± 9.2** ***	294.2 ± 37.9	7.5 ± 2.6** ***	15.4 ± 4.0** ***	−14.4 ± 3.2***

Haemodynamic function was recorded throughout the experimental protocol. The data shown are for the end of the equilibration period prior to ischaemia and after 60 min reperfusion. Pre‐ischaemic data are shown as average of all the hearts of this series of experiments.

* *P* < 0.05 significantly different from control;

** *P* < 0.05 significantly different from 8‐Br of the corresponding experimental series;

*** *P* < 0.05 significantly different from initial (end of equilibration) value of the same group of hearts.

**Table 2:** Effects of 6‐Bnz and CPT on haemodynamic function recovery during reperfusion

Parameters	LVDP (mmHg)	HR (beat·min^−1^)	RPP (mmHg·beat·min^−1^)·10^3^	+dP·dt^−1^ (mmHg·s^−1^)·10^2^	−dP·dt^−1^ (mmHg·s^−1^)·10^2^
Pre‐ischaemic equilibration (*n* = 23)	77.6 ± 5.1	277.2 ± 6.1	21.4 ± 1.4	30.7 ± 1.5	−20.4 ± 1.1
Reperfusion					
Control (*n* = 7)	30.1 ± 5.9	228.6 ± 39.5	6.9 ± 1.2	12.0 ± 1.6	−10.3 ± 0.9
6−Bnz (*n* = 5)	23.6 ± 4.2**	240.8 ± 30.4	6.0 ± 1.4**	11.2 ± 1.5**	−9.5 ± 1.4**
CPT (*n* = 5)	30.9 ± 9.9**	248.2 ± 27.5	7.1 ± 2.3**	11.9 ± 2.3**	−10.4 ± 1.5**
6−Bnz + CPT (*n* = 6)	66.2 ± 10.8*	243.2 ± 7.5	16.1 ± 2.7*	22.1 ± 3.9*	−17.1 ± 2.3*

Haemodynamic function was recorded throughout the experimental protocol. The data shown are for the end of the equilibration period prior to ischaemia and after 60 min reperfusion. Pre‐ischaemic data are shown as average off all the hearts of this series of experiments.

* *P* < 0.05, significantly different from control;

** *P* < 0.05, significantly different from 6‐Bnz + CPT.

### Heart perfusion protocols

After a 25 min period of equilibration followed by pre‐ischaemic interventions, global normothermic ischaemia (37°C) was induced for 30 min, and then normothermic perfusion was reinstated for 2 h.

**Figure 1:**
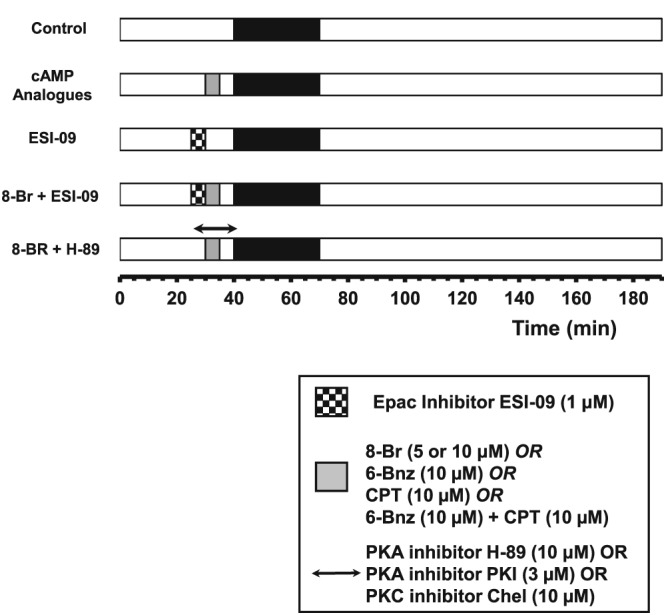
Outline of the experimental protocols. *Experimental Series 1.* Hearts were perfused with an activator of both PKA and Epac (8‐Br, 5 μM), an inhibitor of PKA (H‐89) and Epac (ESI‐09). Groups of hearts: Control, 8‐Br, 8‐Br + H‐89 and 8‐Br + ESI‐09. *Experimental Series 2.* Hearts were perfused with 8‐Br (10 μM), a PKC inhibitor chelerythrine (Chel) and a specific PKA inhibitor peptide PKI. Groups of hearts: Control, 8‐Br, 8‐Br + PKI and 8‐Br + Chel. *Experimental Series 3.* Hearts were perfused with a PKA activator (6‐Bnz) and an Epac activator (CPT). Groups of hearts: Control, 6‐Bnz, CPT and 6‐Bnz + CPT.

To investigate the cardioprotective effects of 8‐Br against I/R injury, hearts were randomly distributed between control or interventions groups (six to seven hearts per group, Figure 1). These hearts were treated with either 5 or 10 μM 8‐Br for 5 min followed by 5 min washout immediately prior to index ischaemia (Figure 1). We used 5 μM 8‐Br for optimization and in our early experiments in Series 1. However, in Series 2 performed 2 years later, the hearts were more vulnerable to I/R injury compared with Series 1 as shown by higher infarct size (70.1 ± 5.2% vs. 45.5 ± 5.7%, respectively; *P* < 0.05, Figures 5B and 6B). As a result, 5 μM 8‐Br produced a relatively smaller cardioprotective effect. Therefore, the dose was increased to 10 μM, which generated a comparable marked protective effect (see Results). To test the effect of 8‐Br in the presence of an Epac inhibitor, hearts were perfused with 1 μM ESI‐09 for 5 min followed by 5 min perfusion with 5 μM 8‐Br and 5 min washout. In an additional group of experiments, PKA inhibitors, 10 μM of H‐89 or 3 μM of PKI (Glass *et al.,* 1989), were present 5 min before, 5 min during and 5 min after the heart perfusion with 8‐Br. Control hearts were perfused without drugs.

To investigate the effects of the PKA activator 6‐Bnz and the Epac activator CPT alone and in a combination, hearts were randomly distributed between control or interventions groups (five to six hearts per group, Figure 1). In the control group, hearts were perfused for 35 min with no intervention prior to ischaemia. Three other groups of hearts were perfused with either the PKA activator 6‐Bnz (10 μM), the Epac activator CPT (10 μM) or the mixture of these two cAMP analogues (Figure 1). These cAMP analogues were perfused for 5 min followed by 5 min washout.

Additional hearts (six hearts per group) with or without treatment with 8‐Br, 6‐Bnz or CPT were also collected, frozen in liquid nitrogen at the end of the pre‐ischaemic protocol and used to determine PKCδ and PKCε translocation from the cytosol to membrane fraction, VASP phosphorylation or PKA activity.

To investigate the involvement of PKC in mediating the effects associated with PKA and Epac activation by 8‐Br (Christensen *et al.,* 2003), hearts were randomly divided into a control group, a 8‐Br group and a 8‐Br + chelerythrine group (five hearts per group, Figure 1). In the control group, the hearts were perfused for 40 min with no intervention prior to ischaemia. In the other two groups, hearts were perfused with 8‐Br (10 μM) for 5 min in the absence or presence of the non‐selective PKC inhibitor, chelerythrine (10 μM) according to the protocol similar to that with the PKA inhibitors.

Analysis of haemodynamic function recovery, LDH release during reperfusion and infarct size, was performed in all hearts subjected to I/R.

The specific Epac inhibitory activity of ESI‐09 was confirmed previously (Chen *et al.,* 2013). Rehmann H *et al.* (2013) found that ESI‐09 have general protein denaturing properties. However, a recent study (Zhu *et al.,* 2015) has revealed that ESI‐09 inhibits Epac activity with apparent IC_50_ values well below the concentrations (over 25 μM) shown to induce its effect on protein folding.

Previously, we have shown on a similar model of I/R that H‐89 or chelerythrine alone at the concentration of 10 μM had no effect on I/R injury (Khaliulin *et al.,* 2007; Khaliulin *et al.,* 2010). For this reason, the groups of hearts with H‐89 or chelerythrine alone were not included in this study.

### Cardiac injury

LDH activity in the effluent perfusate collected from the hearts of all groups prior to ischaemia and during each 5 min over the first 30 min of reperfusion was determined as described previously (Bergmeyer and Bernt, 1963; Khaliulin *et al.,* 2014). Infarct size was determined as described elsewhere (Pasdois *et al.,* 2007). Briefly, at the end of reperfusion, hearts were stained with 1% of triphenyltetrazolium chloride, frozen at −20°C, sliced into six slices and immersed in 4% formaldehyde. Images of the heart slices were obtained using an HP scanner, and the necrotic and intact areas of each side for each of the heart slices were determined using AlphaEase v5.5 software. The total necrotic and intact area of ventricular myocardium for each heart was calculated. As the entire heart was at risk from global ischaemia, the infarct size was expressed by dividing the sum of necrotic areas by the sum of total slice areas of the six slices to obtain the percentage of necrosis. Infarct size (Figures 5B, 6B and 7C) is expressed as percentage of the whole heart area in order to avoid unwanted variations of the absolute values.

### Data and statistical analysis

The data and statistical analysis in this study comply with the recommendations on experimental design and analysis in pharmacology (Curtis *et al*., 2015). Data are presented as mean ± SEM. The assignment of hearts to different groups was randomized. The raw data were assessed independently by two co‐authors to ensure the correctness of conclusions. Blinding was not used since all the measurements were not subjective but strictly quantitative data. To detect a 25% change at a power 0.8 and α = 0.05 with 8% SD, the effect size required a minimum *n* = 5 animals per group as calculated by the GPOWER programme. The statistical analysis was performed using the software SPSS Statistics, Version 23 (IBM). Statistically significant differences in the LDH activity, haemodynamic function during reperfusion, infarct size, protein expression in Western blots and the PKA kinase activity between the groups were evaluated by one‐way ANOVA followed by Fisher's LSD multiple comparison *post hoc* test. Pre‐ischaemic parameters of haemodynamic function and changes of the haemodynamic parameters during reperfusion compared with the pre‐ischaemic values were assessed by one‐way ANOVA with repeated measures followed by Fisher's LSD test. The limit of statistical significance was *P* < 0.05. The *post hoc* tests were performed if F‐test of variance had achieved the necessary level of statistical significance.

### Materials

The cell‐permeable cAMP analogues: 8‐Br (8‐bromoadenosine‐3′,5′‐cyclic monophosphate, acetoxymethyl ester); 6‐Bnz (N^6^‐benzoyladenosine‐3′,5′‐cyclic monophosphate, acetoxymethyl ester; CPT (8‐(4‐chlorophenylthio)‐2′‐O‐methyladenosine‐3′,5′‐cyclic monophosphate, acetoxymethyl ester); and the Epac inhibitor, ESI‐09 (3‐[5‐(tert.‐butyl)isoxazol‐3‐yl]‐2‐[2‐(3‐chlorophenyl)hydrazono]‐3‐oxopropanenitrile) (Almahariq et al., 2013) were purchased from BioLog Life Science Institute (Bremen, Germany). The PKA inhibitor 14–22 amide (PKI), cell‐permeable, myristoylated was purchased from Merck Millipore (Watford, UK). Other biochemicals were purchased from Sigma (Gillingham, UK), including the PKA inhibitor H‐89 and the PKC inhibitor chelerythrine. General chemicals were purchased from Fischer Scientific (Loughborough, UK) or VWR‐Jencons (Lutterworth, UK).

## Results

### Optimization and validation of the effects of cAMP analogues and inhibitors

The indirect (using isoprenaline) or direct (using 8‐Br) activation of PKA and Epac caused a significant increase in the amplitude of Ca^2+^ transients measured in electrically stimulated and superfused cardiomyocytes (Supplementary Information Fig. S1). Interestingly, 5 μM 8‐Br (activator of both PKA and Epac) increased Ca^2+^ transients to the same extent as the β‐adrenoceptor agonist isoprenaline (10 nM). We have previously shown that this concentration of isoprenaline, in combination with adenosine, produced a powerful cardioprotective effect (Khaliulin *et al.,* 2014). Consequently, 5 μM 8‐Br concentration was chosen for the Experimental Series 1, when this compound was used in combination with the PKA inhibitor, H‐89 or the Epac inhibitor, ESI‐09.

As cAMP is known to activate Epac, in addition to PKA, we studied the role of Epac in mediating cardioprotection. Experiments with H9C2 cells showed that 5 μM 8‐Br (non‐selective activator of PKA and Epac) and 10 μM CPT (selective activator of Epac) increased mRNA expression of the Epac‐target gene, c‐fos, by 32 and 24‐fold respectively (Supplementary Information Fig. S2a) indicating similar activation of Epac by these two cAMP analogues. The 8‐Br‐induced c‐fos expression was completely blocked by the Epac inhibitor ESI‐09 (1 μM), consistent with Epac‐dependent regulation.

Phosphorylation of the PKA substrate VASP, on Ser^157^, was used to compare PKA activation induced by 8‐Br and 6‐Bnz. Stimulation with 5 μM 8‐Br was found to evoke a similar level of VASP phosphorylation as 10 μM of the selective PKA activator, 6‐Bnz (Supplementary Information Fig. S2b).

We measured PKA activity in hearts treated with 6‐Bnz and CPT (Supplementary Information Fig. S3). Treating hearts with CPT did not alter PKA activity. This was consistent with published data indicating specificity of this Epac activator (Chen *et al.,* 2013; Zhu *et al.,* 2015). In contrast, PKA activation by 6‐Bnz was evident in these experiments. These data confirm the selectivity of 6‐Bnz for PKA and inability of the Epac agonist CPT (10 μM) to activate PKA.

Our experiments show that 5 μM 8‐Br induced a similar level of PKA activation as 10 μM 6‐Bnz, which matched the PKA activation induced by 10 nM Iso used in our earlier study (Khaliulin *et al.,* 2014). 8‐Br at a concentration of 5 μM also induced Epac activation to a similar extent as 10 μM CPT. We therefore used these concentrations of the three different activators for evaluation of functional activity in Langendorff‐perfused hearts.

### Pre‐ischaemic haemodynamic effects of cAMP analogues

#### Effects of simultaneous activation of PKA and Epac by 8‐Br

**Figure 2:**
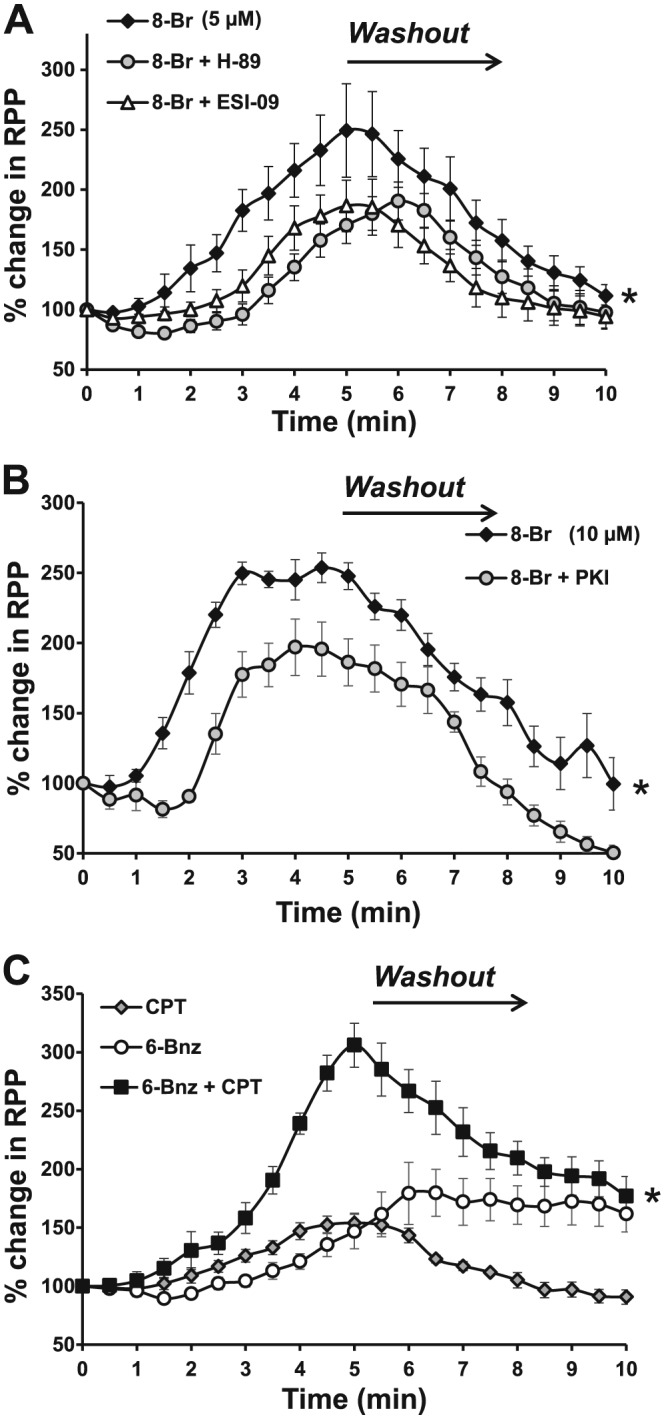
The effect of cAMP analogues on RPP of rat isolated Langendorff‐perfused hearts. The data are presented as percentage of pretreatment values calculated for each heart. Panel A – Changes in RPP induced by heart perfusion with 8‐Br (5 μM) alone or in the presence of 1 μM ESI‐09 or 10 μM H‐89. Groups of hearts: 8‐Br, *n* = 7; 8‐Br + ESI‐09, *n* = 7; 8‐Br + H‐89, *n* = 6. *P < 0.05, significant effect of inhibitor (H‐89 or ESI‐09). Panel B – Changes of RPP induced by heart perfusion with 8‐Br (10 μM) alone or in the presence of 3 μM PKI. Groups of hearts: 8‐Br, *n* = 7; PKI, *n* = 5; 8‐Br + PKI, *n* = 5. Panel C – Changes of RPP induced by the heart perfusion with 6‐Bnz (10 μM) and CPT (10 μM) alone or in combination. Groups of hearts: 6‐Bnz, *n* = 5; CPT, *n* = 5; 6‐Bnz + CPT, *n* = 6. *P < 0.05, significantly different from the combination of 6‐Bnz and CPT.

Perfusion of hearts with 5 μM 8‐Br resulted in a marked increase in RPP, almost 250% of the pretreatment value, by the end of the 5 min perfusion. Switching the perfusion to normal KH (washout) reversed the changes in RPP back to the initial value, by the end of the washout period (Figure 2A). The increased haemodynamic function induced by 8‐Br was a result of increased LVDP since no significant change in HR was observed. When hearts were perfused with 10 μM 8‐Br, the changes in RPP were similar to those treated with 5 μM 8‐Br (Figure 2B). ESI‐09 (1 μM), H‐89 (10 μM) and PKI (3 μM) inhibited, to almost the same extent, the positive inotropic effect of 8‐Br (Figure 2A–B).

#### Effects of PKA and/or Epac activation using 6‐Bnz and CPT

Perfusion of isolated hearts for 5 min, with 10 μM 6‐Bnz brought about a slow LVDP‐related increase in RPP (Figure 2C). This effect reached a maximum during the washout period. CPT alone (10 μM) also induced a positive inotropic effect with a rise in RPP at the end of the 5 min perfusion with the compound. This was significantly lower than in 8‐Br‐treated hearts at the same time point of the experiment (*P* < 0.05). Interestingly, the positive inotropic effect of CPT was readily reversible with washing. Perfusion of hearts with the mixture of 10 μM 6‐Bnz and 10 μM CPT led to a very sharp rise in RPP which was twofold higher than in hearts treated with either 6‐Bnz or CPT alone, at the end of the 5 min perfusion (Figure 2C).

### The effect of heart perfusion with 8‐Br on activation of PKCδ and PKCε

**Figure 3:**
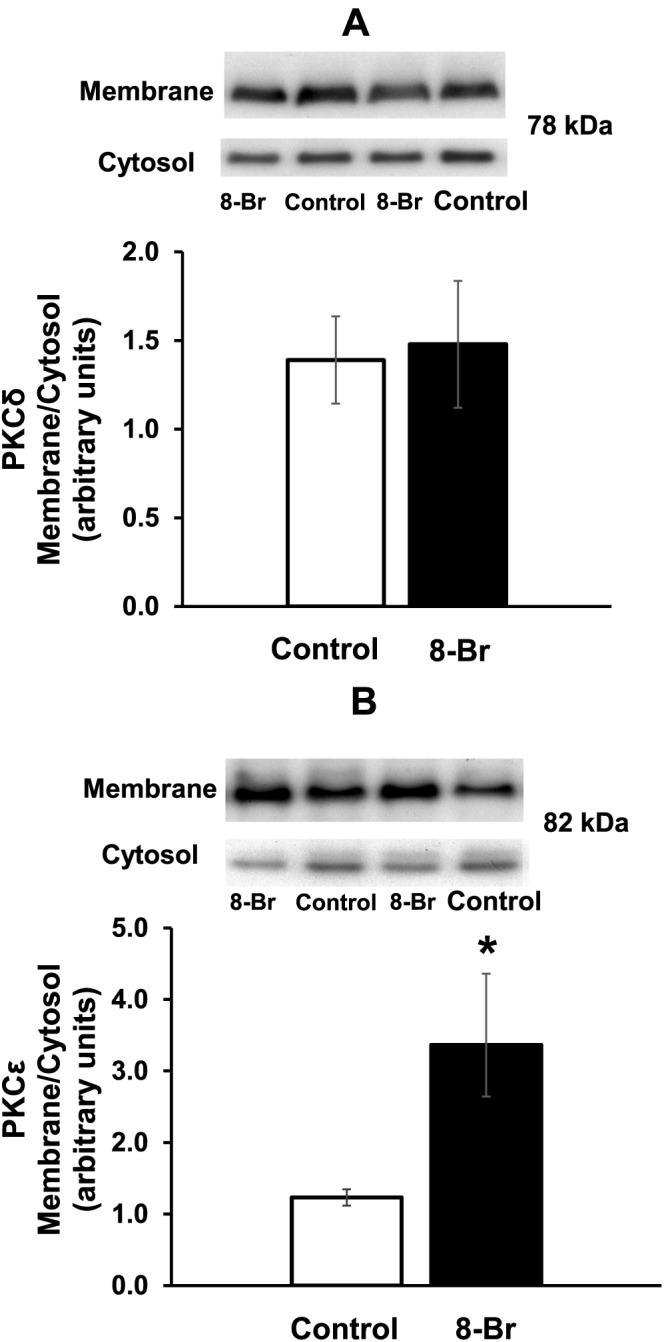
The effect of 8‐Br on translocation of PKCδ and PKCε from the cytosol to membrane fraction. PKC translocation, reflecting its activation, was assessed by Western blots and expressed as a ratio of the optical density of the membrane‐to‐cytosol fraction. Groups of hearts: control, *n* = 6; 8‐Br, *n* = 6. Panel A – 8‐Br (5 μM) had no effect on PKCδ translocation. Panel B – 8‐Br (5 μM) promoted PKCε translocation. *P < 0.05, significantly different from control.

Perfusion of hearts with 5 μM 8‐Br had no effect on PKCδ translocation (activation) from the cytosol to the membrane fraction (Figure 3A). However, there was a significant increase in PKCε activation as shown by a nearly threefold increase in the membrane‐to‐cytosol ratio, as assessed by optical density, in 8‐Br‐treated hearts (Figure 3B). Equal protein loading was confirmed by Ponceau‐S staining (Supplementary Information Fig. S4)

### The cardioprotective effect of simultaneous activation of PKA and Epac using 8‐Br

Hearts were perfused with 5 μM 8‐Br for 5 min with or without an Epac inhibitor ESI‐09 or PKA inhibitors H‐89 and PKI (see the Methods for details). The drugs were washed out for 5 min before inducing a 30 min global ischaemia followed by 2 h reperfusion.

#### Recovery of haemodynamic function

**Figure 4:**
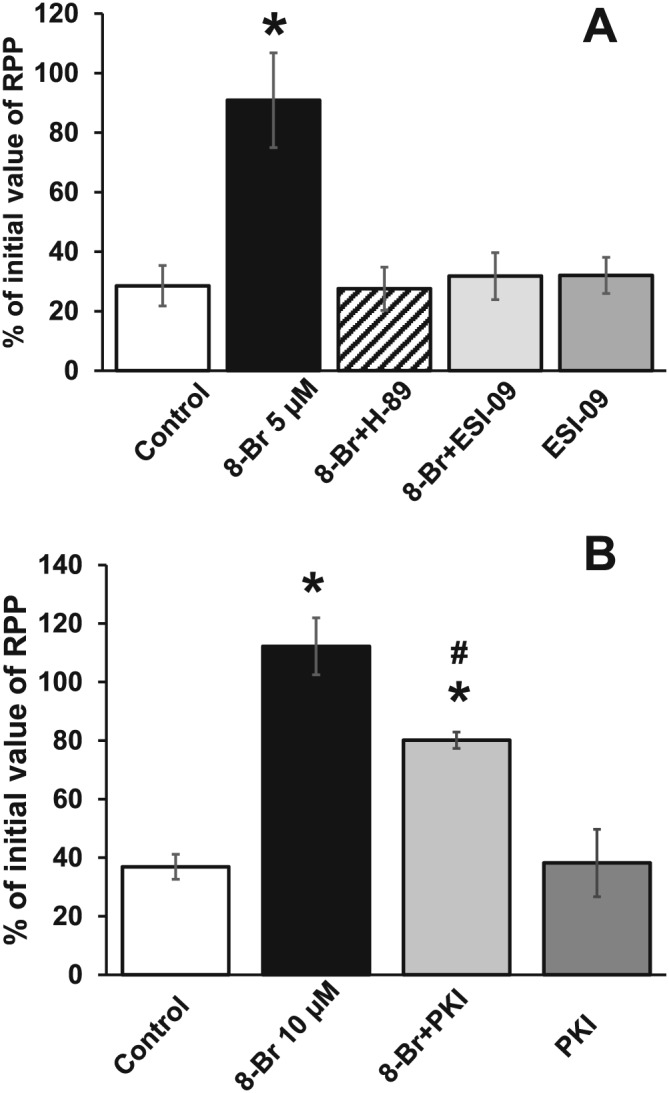
The effect of 8‐Br with or without ESI‐09, H‐89 and PKI on RPP recovery after I/R. For clarity, RPP is expressed as percentage of the initial value measured at the end of equilibration period prior to any intervention. Panel A – Groups of hearts: Control, *n* = 7; 8‐Br, *n* = 7; ESI‐09, *n* = 7; 8‐Br + ESI‐09, *n* = 7; 8‐Br + H‐89, *n* = 6. Pretreatment of hearts with 8‐Br (5 μM) fully restored RPP after 30 min global ischaemia and 60 min reperfusion, but ESI‐09 (1 μM) and H‐89 (10 μM) abolished this effect. Perfusion of hearts with ESI‐09 alone had no effect on the RPP recovery. Panel B – Groups of hearts: Control, *n* = 7; 8‐Br, *n* = 7; PKI, *n* = 5; 8‐Br + PKI, *n* = 5. Pretreatment of hearts with 8‐Br (10 μM, *n* = 7) fully restored RPP, but PKI (3 μM, *n* = 5) inhibited this effect. Perfusion of hearts with PKI alone had no effect on the RPP recovery. *P < 0.05, significantly different from control; #*P* < 0.05, significantly different from 8‐Br.

Recovery of haemodynamic function during reperfusion in the control hearts, in hearts treated with 8‐Br (with or without ESI‐09, H‐89 and PKI) and in hearts perfused with ESI‐09 or PKI alone is presented in Table 1 and Figure 4. There was poor functional recovery in the control, untreated hearts, as measured by the developmental rate of contraction (+dP/dt) and relaxation (−dP/dt) and LVDP. These indices recovered to less than 50% of the corresponding pre‐ischaemic values. The RPP at the end of 1 h reperfusion was markedly lower compared with the baseline (Table 1, Figure 4). This effect was largely due to changes in LVDP as there was no significant change in computed HR. Pretreatment with either ESI‐09 (1 μM) or PKI (3 μM) did not affect the functional recovery. Pretreatment of hearts with 5 μM 8‐Br prior to ischaemia markedly improved functional recovery during reperfusion with +dP/dt, LVDP and RPP showing recovery to >90% of the pre‐ischaemic values (Table 1, Figure 4A). In hearts pretreated with 10 μM 8‐Br, there was also a full recovery of RPP (Table 1, Figure 4B). The improved functional recovery in hearts treated with 8‐Br was prevented by the pretreatment with either a PKA inhibitor (10 μM H‐89) or Epac inhibitor (1 μM ESI‐09) (Table 1, Figure 4A). This was also confirmed using a selective PKA inhibitor peptide PKI, which partly reduced 8‐Br‐induced improvement of the recovery of haemodynamic function (Table 1, Figure 4B).

#### Cardiac injury

**Figure 5:**
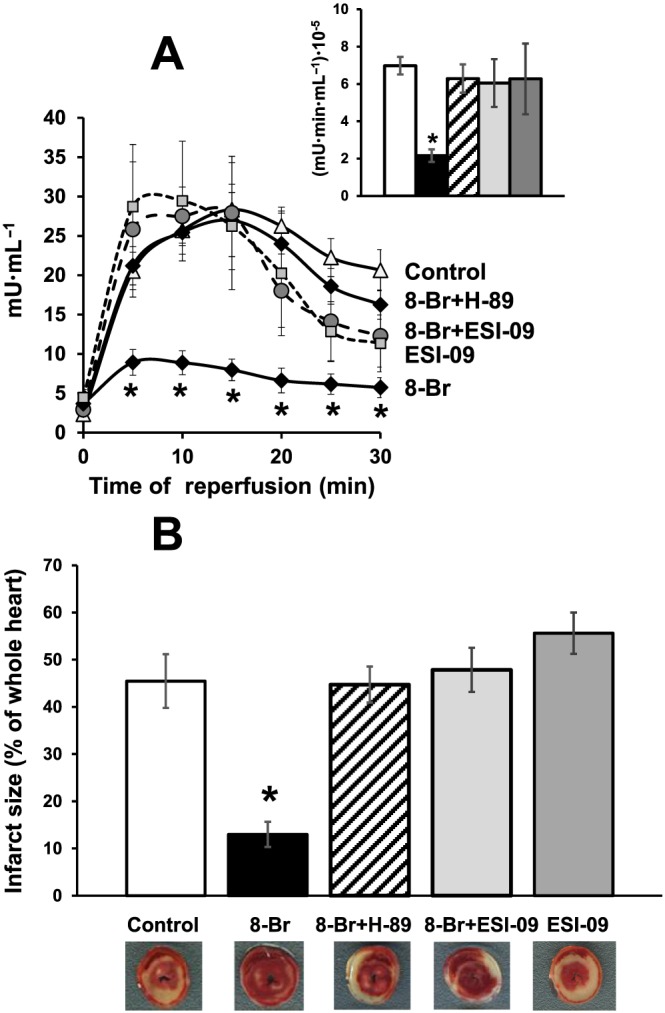
The effect of 8‐Br with or without ESI‐09 and H‐89 on cardiac injury following I/R. Groups of hearts: Control, *n* = 7; 8‐Br, *n* = 7; ESI‐09, *n* = 7; 8‐Br + ESI‐09, *n* = 7; 8‐Br + H‐89, *n* = 6. Panel A – Activity of LDH in the effluent perfusate was reduced by pretreatment of hearts with 8‐Br (5 μM), but ESI‐09 (1 μM) and H‐89 (10 μM) abolished this effect. Insert – Mean area under the curve reflecting the total release of LDH. The column‐fills in the insert correspond to the column‐fills in Panel B. Panel B – Infarct size was calculated as percentage of the whole heart area. Infarct size during reperfusion was reduced by pretreatment of hearts with 8‐Br (5 μM), but ESI‐09 (1 μM) and H‐89 (10 μM) abolished this effect. ESI‐09 alone had no effect on cardiac injury. Representative heart slices with infarcted myocardium (pale white area) are shown under the corresponding columns. *P < 0.05, significantly different from control.

**Figure 6:**
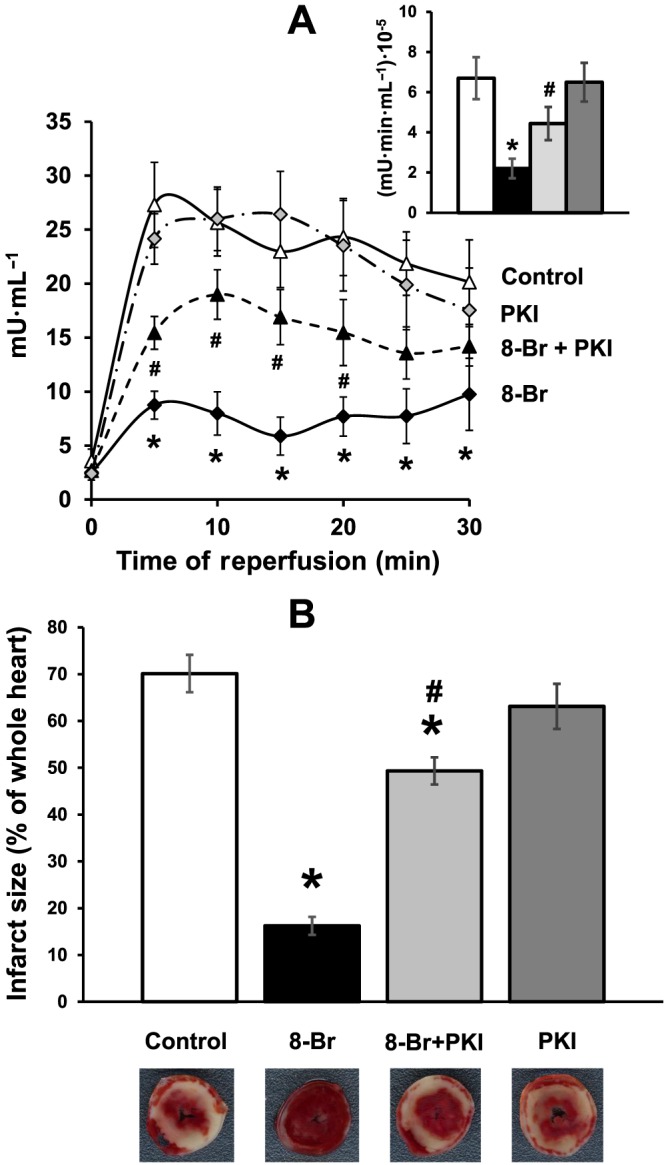
The effect of 8‐Br with or without PKI on cardiac injury following I/R. Groups of hearts: Control, *n* = 7; 8‐Br, *n* = 7; PKI, *n* = 5; 8‐Br + PKI, *n* = 5. Panel A – LDH activity in the effluent perfusate was reduced by pretreatment of hearts with 8‐Br (10 μM) during reperfusion, but PKI (3 μM) inhibited this effect. Insert – Mean area under the curve reflecting the total release of LDH. The column‐fills in the insert correspond to the column‐fills in Panel B. Panel B – Infarct size was calculated as percentage of the whole heart area. Infarct size was significantly reduced by 8‐Br (10 μM), but PKI (3 μM) inhibited this effect. PKI alone had no effect on cardiac injury. Representative heart slices with infarcted myocardium (pale white area) are shown under the corresponding columns. *P < 0.05, significantly different from control (*n* = 7); # *P* < 0.05, significantly different from 8‐Br.

The excellent recovery in cardiac function following I/R in hearts pretreated with 8‐Br was also associated with a significant decrease in cardiac injury as measured by LDH release and infarct size (Figures 5 and 6). 8‐Br reduced LDH activity by threefold and infarct size by 3.5‐fold. Consistent with effects on functional recovery, both ESI‐09 and H‐89 abolished (Figure 5), and PKI inhibited this effect (Figure 6). This was manifested through increased LDH activity and infarct size during reperfusion. Neither ESI‐09 nor PKI alone had any effect on LDH release or infarct size during reperfusion.

### The cardioprotective effects of activating either PKA or Epac

In these experiments, hearts were perfused with either 6‐Bnz or CPT prior to I/R to investigate the effect of PKA and Epac activation respectively.

#### Recovery of haemodynamic function

**Figure 7:**
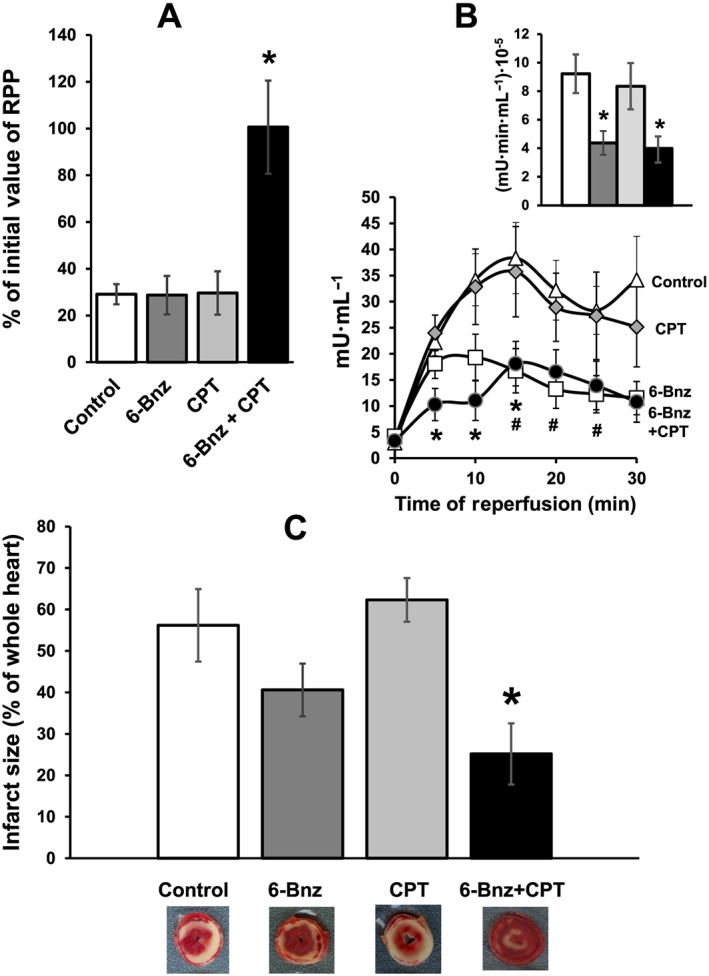
The effect of 6‐Bnz or CPT on RPP recovery and cardiac injury following I/R. Groups of hearts: Control, *n* = 7; 6‐Bnz + CPT, *n* = 6; CPT, *n* = 5; 6‐Bnz, *n* = 5. Panel A – RPP is expressed as percentage of the initial value measured at the end of equilibration period prior to any intervention. Neither 6‐Bnz (10 μM) nor CPT (10 μM) had any effect on RPP recovery after 30 min ischaemia and 60 min of reperfusion, but the mixture of 6‐Bnz and CPT significantly improved the recovery. Panel B – LDH activity in the effluent perfusate was reduced by pretreatment of hearts with 6‐Bnz (10 μM) starting from 15 min of reperfusion; pretreatment of hearts with CPT (10 μM) had no effect, but the mixture of 6‐Bnz and CPT significantly reduced LDH activity starting from the beginning of reperfusion. Insert – Mean area under the curve reflecting the total release of LDH. The column‐fills in the insert correspond to the column‐fills in Panels A and C. Panel C – Infarct size was calculated as percentage of the whole heart area. Neither 6‐Bnz nor CPT alone reduced infarct size whilst the mixture of 6‐Bnz and CPT significantly reduced infarct size. Representative heart slices with infarcted myocardium (pale white area) are shown under the corresponding columns. *P < 0.05, 6‐Bnz + CPT significantly different from control; #*P* < 0.05, 6‐Bnz significantly different from control.

Post‐ischaemic recovery of haemodynamic function in control hearts and in hearts pretreated with the selective PKA activator 6‐Bnz and the Epac specific activator CPT is presented in Figure 7A and Table 2. Neither perfusion of hearts with 10 μM 6‐Bnz nor with 10 μM CPT alone improved recovery of haemodynamic parameters during reperfusion, compared with the control values. However, pre‐ischaemic perfusion of hearts with the mixture of these two cAMP analogues resulted in a complete recovery of +dP/dt, −dP/dt, LVDP and RPP.

#### Cardiac injury

As shown in Figure 7B, pretreatment of hearts with CPT had no effect on LDH activity in the effluent collected during reperfusion. Meanwhile, 6‐Bnz transiently reduced LDH activity, which became significant compared with control after 15 min of reperfusion. Perfusion of hearts with the mixture of 6‐Bnz and CPT resulted in a significant reduction of LDH throughout reperfusion (Figure 7B).

Although there was a trend for hearts pretreated with 6‐Bnz to have a smaller infarct size compared with control, this was not statistically significant. Pretreatment with CPT did not affect infarct size. However, perfusion of hearts with the mixture of 6‐Bnz and CPT led to a significant, nearly twofold reduction of infarct size (Figure 7C).

### The effect of PKC inhibition on cardioprotection mediated by simultaneous activation of PKA and Epac

In this set of experiments, hearts were perfused with 10 μM 8‐Br for 5 min with or without the non‐selective PKC inhibitor, chelerythrine (10 μM).

#### Recovery of haemodynamic function

Functional recovery in control hearts and in hearts treated with 8‐Br, with or without chelerythrine (Experimental series 2), is presented in Table 1. The RPP in hearts pretreated with 8‐Br reached a value threefold higher than in the control group, after 1 h of reperfusion. Addition of chelerythrine did not affect the recovery of RPP in the 8‐Br‐treated hearts. However, recovery of ‐dP/dt in hearts perfused with chelerythrine and 8‐Br was significantly less than in the hearts perfused with 8‐Br alone.

#### Cardiac injury

**Figure 8:**
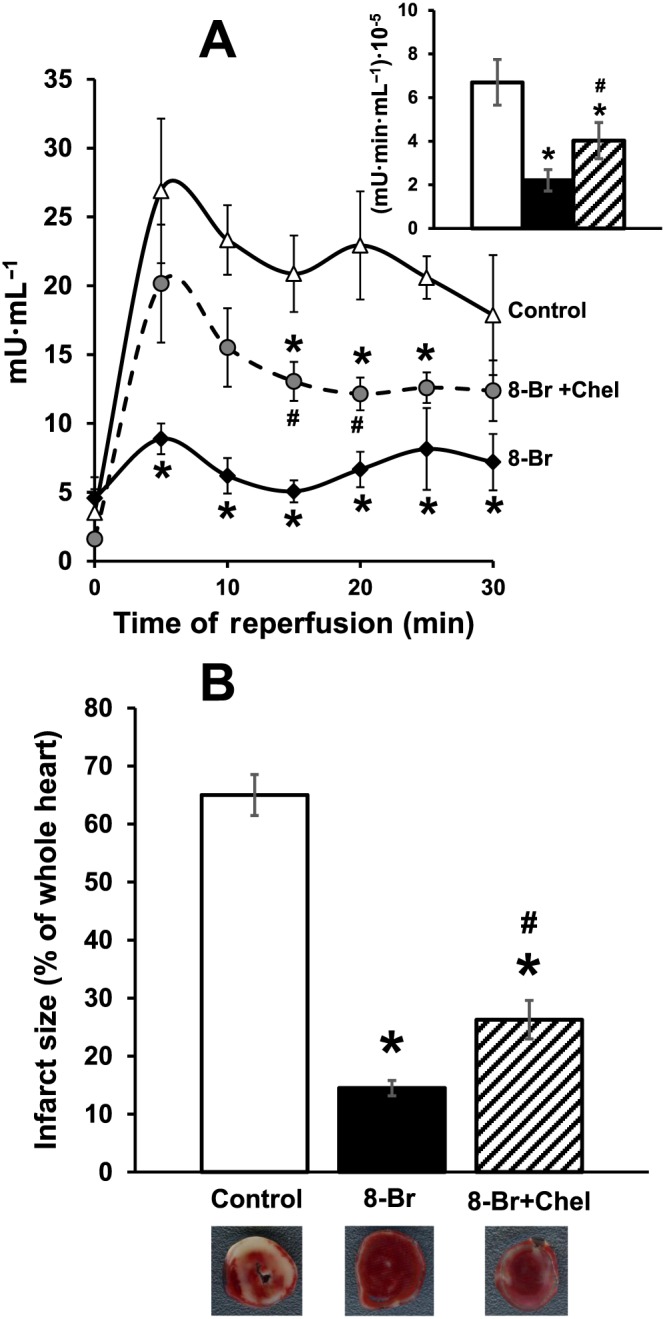
The effect of 8‐Br and chelerythrine (Chel) on cardiac injury following I/R. Cardiac injury was assessed by measuring LDH activity and infarct size. Groups of hearts: Control, *n* = 7; 8‐Br, *n* = 7; 8‐Br + Chel, *n* = 5. Panel A – 8‐Br (10 μM) reduced LDH activity in the effluent perfusate during reperfusion, but chelerythrine (10 μM) reversed this effect. Panel B – 8‐Br (10 μM) significantly reduced infarct size during reperfusion, but chelerythrine (10 μM) attenuated this effect. *P < 0.05, significantly different from control; #*P* < 0.05, significantly different from 8‐Br.

Pretreatment of hearts with 10 μM 8‐Br reduced LDH activity (Figure 8A) and infarct size by 4.5‐fold (Figure 8B) confirming a strong protective effect. Adding 10 μM chelerythrine during the 8‐Br pretreatment reduced this effect.

## Discussion

The availability of cell‐permeable cAMP analogues that can selectively activate either PKA or Epac or both has provided a useful tool to study the contribution of PKA and Epac to the cardioprotection against I/R injury. We have used these analogues to demonstrate that optimal cardioprotection can only be achieved by simultaneous activation of both enzymes. Our results also indicate that cardioprotection induced by simultaneous activation of both PKA and Epac using 8‐Br is mediated, at least in part, by PKC.

In contrast to this study, earlier investigations of the cardioprotective effects of cAMP were linked to activation of adrenoceptors (Lochner *et al.,* 1999; Khaliulin *et al.,* 2010). To the best of our knowledge, this is the first report demonstrating a β‐adrenoceptor‐independent cardioprotective effect of cAMP. This may provide a therapeutic advantage as the sensitivity of β‐adrenoceptors could be impaired in particular clinical settings (see Introduction).

Another interesting finding of this work is that activation of either PKA or Epac using selective activators was sufficient to induce a clear inotropic effect in the perfused heart and was additive when these enzymes were activated simultaneously. Therefore, the role of PKA and Epac activation in regulating cardiac function and in cardioprotection is likely to be due to parallel, mutually additive effects of PKA and Epac or due to an interaction between these two signalling pathways.

### Activation of both PKA and Epac is necessary for maximal inotropic effect of cAMP

Perfusion of hearts with specific activators of either PKA or Epac brought about an increase in cardiac pump function (Figure 2). The positive inotropic effect of PKA is not surprising (Bers, 2007). PKA has been shown to stimulate excitation‐contraction coupling by phosphorylating and modulating activity of a variety of myocardial proteins, including the L‐type Ca^2+^ channel, ryanodine receptor (RyR2), phospholamban, Ca^2+^/calmodulin‐dependent protein kinase II (CaMKII), sarcoplasmic reticulum Ca^2+^ ATPase and troponin I, ensuring mobilization of energy resources, up‐regulation of Ca^2+^ cycling and increases in contractility and lusitropy. However, the finding that Epac stimulates contraction was interesting as it was reported to mediate its actions independently of PKA pathways (Pereira *et al.,* 2015). Epac activation mediated by stimulation of β‐adrenoceptors has been linked to sarcoplasmic reticulum Ca^2+^ release. It has been shown to activate CaMKII resulting in phosphorylation of RyR2 and phospholamban (Hothi *et al.,* 2008; Oestreich *et al.,* 2009). An Epac‐induced increase in contractility has also been shown in experiments on rat and mouse cardiomyocytes (Metrich *et al.,* 2010). The availability of parallel Ca^2+^ regulatory pathways associated with Epac and PKA, which independently trigger inotropic effects, may explain the additive inotropic effect of the mixture of CPT and 6‐Bnz observed in our experiments.

### Simultaneous activation of PKA and Epac confers cardioprotection mediated by PKC

The cardiac effects associated with cAMP signalling depends on a number of factors including but not limited to the dose, timing and duration of intervention and the balance between activation of PKA and Epac‐related pathways. For example, sustained stimulation of β‐adrenoceptors can lead to mitochondria‐induced apoptosis, contractile dysfunction, cardiac hypertrophy, arrhythmias, cell death and, eventually, heart failure (Dorn *et al.,* 2000; Appukuttan *et al.,* 2012; Rau *et al.,* 2015).

In contrast to sustained β‐adrenoceptor stimulation, a moderate pre‐ischaemic stimulation of the same receptors is cardioprotective. Earlier data show that PKA activation induced by stimulation of β‐adrenoceptors, triggers an ischaemic preconditioning (IP)‐like effect (Lochner *et al.,* 1999) and that repeated stimulation with noradrenaline or isoprenaline mimics IP (Asimakis *et al.,* 1994). Previously, we found that temperature preconditioning induced by a few short‐term episodes of hypothermic and normothermic perfusion (Khaliulin *et al.,* 2007) is also mediated by PKA activation (Khaliulin *et al.,* 2010).

Consistent with a role of PKA in cardioprotection, the PKA activator 6‐Bnz significantly reduced LDH activity in the effluent perfusate during reperfusion (Figure 7B). One possible explanation for the cardioprotective effect of PKA activation could be the fact that activation of this kinase by the β‐adrenoceptor agonist isoprenaline triggers glycogen depletion (Khaliulin *et al.,* 2010). This may result in reduced accumulation of lactate, H^+^, Na^+^ and Ca^2+^ (Cross *et al.,* 1996), contributing to the cardioprotection observed (Halestrap and Pasdois, 2009). A recent study has also linked the depletion of myocardial glycogen prior to ischaemia to cardioprotection via less dissociation of hexokinase II from mitochondria, which results in MPTP inhibition (Pasdois *et al.,* 2013). The signalling pathways and targets that can potentially be involved in the cardioprotective effect of PKA may include the reduction of IKK/IκB phosphorylation (Zhang *et al.,* 2013), phosphorylation of GSK‐3β (Gomez *et al.,* 2008; Juhaszova *et al.,* 2004) and regulation of phosphodiesterase (Omori and Kotera, 2007).

However, the protective effect of PKA activation in our experiments was small and was not supported by a reduction of infarct size (Figure 7C) or improvement in functional recovery (Table 2, Figure 7A). This finding does not appear to be due to the extent of PKA activation by 6‐Bnz as 8‐Br, which produces comparable activation of PKA (Supplementary Information Fig. S2b), confers marked cardioprotection. This clearly implies involvement of additional cAMP‐sensitive pathways, which may include Epac.

Consistent with the results reported by others (Duquesnes *et al.,* 2010) who used a similar model of I/R, perfusion of hearts with the Epac agonist CPT did not show a clear protective effect. Nonetheless, we found that perfusion of hearts with the mixture of the specific PKA activator 6‐Bnz and the Epac activator CPT resulted in relatively very high haemodynamic functional recovery (Figure 7A and Table 2), significantly reduced LDH release and infarct size during reperfusion (Figure 7B–C). Similarly, 8‐Br, which simultaneously activates PKA and Epac (Christensen *et al.,* 2003), markedly improved the recovery of haemodynamic function (Table 1, Figure 4) and prevented I/R injury (Figures 5 and 6) implying that both PKA and Epac are involved during cAMP‐induced cardioprotection. This conclusion is also supported by the fact that both PKA inhibitors (H‐89 and PKI) and the Epac inhibitor (ESI‐09), which appeared to have no effect on haemodynamic function recovery or I/R injury by themselves [see (Khaliulin *et al.,* 2010), Table 1 and Figures 4, 5, 6], attenuated the cardioprotective effect of 8‐Br.

Recent work using neonatal rat isolated cardiomyocytes showed that stimulation of glucagon‐like peptide‐1 receptor by exendin‐4 mediated a cytoprotective effect, which was associated with both PKA and Epac‐dependent pathways (Mangmool *et al.,* 2015). Our results, however, do not suggest a simple additive cardioprotective effect of PKA and Epac but a coordinated synergistic action of these two cAMP‐related pathways, particularly considering the absence of any protective effect of Epac activation alone and a relatively weak cardioprotective effect of 6‐Bnz‐induced PKA activation. Obviously, the Epac activation represents a necessary signalling link that magnifies the protective effect of PKA through mechanisms additional to PKA signalling pathways or by enhancing the activation of pathways common to both PKA and Epac. The latter is likely to include PKCε activation, as we discuss below. A synergistic action has also been reported in vascular smooth muscle cells where PKA and Epac synergize to inhibit cell proliferation (Hewer *et al.,* 2011).

Interestingly, our previous work demonstrated that stimulation of β‐adrenoceptors by isoprenaline only induced a mild cardioprotective effect (Khaliulin *et al.,* 2010). This could be because PKA is more sensitive to cAMP than Epac (Bos, 2003). However, when this was followed by PKC activation, the protection was marked (Khaliulin *et al.,* 2010).

Importantly, our results demonstrate that PKC is also involved in the 8‐Br‐induced cardioprotective effect since the non‐selective PKC inhibitor chelerythrine partly blocked the effect of this cAMP analogue (Figure 8A–B, Table 1). 8‐Br promoted translocation of PKCε but not PKCδ from the cytosol to membrane fraction of myocytes indicating a possible role of PKCε in cAMP‐induced cardioprotection. We have previously identified PKC as a critical component of cAMP‐induced cardioprotection (Khaliulin *et al.,* 2010). We found that the PKA inhibitor H‐89, which has little effect on PKC activity (Bain *et al.,* 2007), was able to prevent both PKC activation induced by temperature preconditioning and cardioprotection. These results imply that PKA activation triggers PKC activation in the signalling pathway of temperature preconditioning (Khaliulin *et al.,* 2010). In fact, the cAMP‐induced activation of PKCε may occur downstream to both Epac and PKA activation. Cazorla *et al*. have shown that Epac activates phospholipase C, which hydrolyzes phosphatidylinositol bisphosphate to produce diacylglycerol and inositol triphosphate (IP_3_), leading to PKC activation or IP3 receptor‐dependent Ca^2+^ release (Cazorla *et al.,* 2009). Furthermore, Li *et al*. have recently shown that the β‐adrenoceptor/Epac/PLC pathway may specifically activate PKCε (Li *et al.,* 2015). PKA, in turn, can activate PKC *via* increased ROS production (Novalija *et al.,* 2003) and [Ca^2+^]_i_ either by direct Ca^2+^‐dependent activation or via a G‐protein activated by Ca^2+^‐dependent phospholipase C (Dekker *et al.,* 1999). Hence, it is logical to suggest that PKCε may serve as a connecting link between the PKA and Epac signalling pathways in the cAMP‐induced cardioprotection. This hypothesis, however, requires further investigation.

### Study limitations

Epac‐mediated effects can be attributed to either Epac1 or Epac2 or both. We did not study the role of the two isoforms in our work but assessed the overall effect of Epac activation. The two isoforms are believed to have different sub‐cellular localization, expression and function (Okumura *et al.,* 2014). This has been widely investigated within the mouse heart (Schmidt *et al.,* 2013). However, a recent study demonstrated that both Epac isoforms are present within mitochondria isolated from adult rat myocytes (Wang *et al.,* 2016). Current findings using rat hearts have implied that the cardioprotective effect induced by urocortin‐1 is mediated by activation of Epac2 (Calderon‐Sanchez *et al.,* 2016) and that Epac2 inhibition promotes cardiac arrhythmias (Yang *et al.,* 2016). Nonetheless, in the absence of direct evidence, a role for the Epac1 isoform cannot be excluded. The generation and availability of selective Epac1 and Epac2 inhibitors will provide tools to investigate the role of individual Epac isoforms in the cardioprotective effect of cAMP in future studies.

In conclusion, the results of our study, taken together, demonstrated for the first time that activation of either PKA or Epac alone, prior to ischaemia, produced little or no cardioprotection whereas simultaneous activation of both enzymes conferred optimal and marked protection against I/R injury. This effect was significantly attenuated in the presence of selective inhibitors of PKA and Epac. Activation of PKA and Epac triggers PKC activation. Inhibition of PKC can reduce the cardioprotective effect of cAMP implicating PKC in the coordinated cardioprotective effect of simultaneous activation of PKA and Epac. The additive effect of PKA and Epac activation is also manifested in their positive inotropic effects.

## Author contributions

I.K. and M.S.S. conceived and designed the experiments. I.K., M.S.S., M.B. and A.F.J. secured funding. I.K., M.B., Z.D. and R.A. performed the experiments. I.K., M.B., Z.D. and R.A. analysed the data. I.K. and M.SS. wrote the manuscript. I.K., M.S.S., A.F.J. and J.L.J. provided critical discussion, editing and final approval of the manuscript.

## Conflict of interest

The authors declare no conflicts of interest.

## Declaration of transparency and scientific rigour

This Declaration acknowledges that this paper adheres to the principles for transparent reporting and scientific rigour of preclinical research recommended by funding agencies, publishers and other organisations engaged with supporting research.

## Supporting information


**Figure S1** Isoprenaline (10 nM) and 8‐Br (5 μM) increased amplitude of Ca^2+^ transients in cardiomyocytes to a similar extent. Groups of cells: Isoprenaline (Iso, *n* = 9); 8‐Br (*n* = 7). Panel A – Representative recording of Ca^2+^ transient in a cardiomyocyte superfused with Iso for 5 min followed by 5 min washout. Panel B – Representative recording of Ca^2+^ transients in a cardiomyocyte superfused with 8‐Br for 5 min followed by 5 min washout. Panel C – Value of increase in the amplitude of Ca^2+^ transients in cardiomyocytes treated with isoprenaline and 8‐Br.
**Figure S2** c‐fos gene expression in H9C2 cells treated with Epac activators and inhibitor (panel A) and comparison of VASP phosphorylation by 8‐Br and 6‐Bnz (panel B). Panel A – c‐fos gene expression measured in H9C2 cells incubated at 37°C for 7 h with either 5 μM 8‐Br, 10 μM CPT or 5 μM 8‐Br + 1 μM ESI‐09. The gene expression was calculated as fold change relative to Control. The level of gene expression in the control group was set as 1. Panel B – a Western blot for phospho‐VASP of the lysate of rat heart treated with 8‐Br (5 μM) and 6‐Bnz (10–100 μM). Control – a lysate of untreated Langendorff‐perfused rat heart. pVASP + VE Control – a lysate of rat smooth muscle cells treated with 25 μM forskolin used as a positive control for PKA activation.
**Figure S3** PKA activity in hearts perfused with 10 μM 6‐Bnz or 10 μM CPT. The hearts were freeze‐clamped at the end of the pre‐ischaemic protocol, outlined in the Methods, and powdered under liquid nitrogen. PKA activity was measured using an ELISA‐based PKA kinase activity assay kit (Abcam) in 3 hearts of each of control, 6‐Bnz and CPT groups. The Table shows PKA activity (ΔOD·μg crude protein^−1^) of each heart.
**Figure S4** PVDF membranes containing proteins of the membrane and the cytosol fractions stained with Ponceau‐S for 2 min. The samples of control hearts and hearts treated with 8‐Br (*n* = 6 in each group) were alternated on the gel and transferred on the membrane. The figure confirms an even loading of the proteins of different samples.
