# Supplementary material for: Functional and cardioprotective effects of simultaneous and individual activation of protein kinase A and Epac
Source: Br J Pharmacol. 2017 Feb 14;174(6):438–53. doi: 10.1111/bph.13709 (PMC5323515; doi:10.1111/bph.13709)
Supplement: Supplementary file 1 — Figure S1 Isoprenaline (10 nM) and 8‐Br (5 μM) increased amplitude of Ca2+ transients in cardiomyocytes to a similar extent. Groups of cells: Isoprenaline (Iso, n = 9); 8‐Br (n = 7). Panel A – Representative recording of Ca2+ transient in a cardiomyocyte superfused with Iso for 5 min followed by 5 min washout. Panel B – Representative recording of Ca2+ transients in a cardiomyocyte superfused with 8‐Br for 5 min followed by 5 min washout. Panel C – Value of increase in the amplitude of Ca2+ transients in cardiomyocytes treated with isoprenaline and 8‐Br. Figure S2 c‐fos gene expression in H9C2 cells treated with Epac activators and inhibitor (panel A) and comparison of VASP phosphorylation by 8‐Br and 6‐Bnz (panel B). Panel A – c‐fos gene expression measured in H9C2 cells incubated at 37°C for 7 h with either 5 μM 8‐Br, 10 μM CPT or 5 μM 8‐Br + 1 μM ESI‐09. The gene expression was calculated as fold change relative to Control. The level of gene expression in the control group was set as 1. Panel B – a Western blot for phospho‐VASP of the lysate of rat heart treated with 8‐Br (5 μM) and 6‐Bnz (10–100 μM). Control – a lysate of untreated Langendorff‐perfused rat heart. pVASP + VE Control – a lysate of rat smooth muscle cells treated with 25 μM forskolin used as a positive control for PKA activation. Figure S3 PKA activity in hearts perfused with 10 μM 6‐Bnz or 10 μM CPT. The hearts were freeze‐clamped at the end of the pre‐ischaemic protocol, outlined in the Methods, and powdered under liquid nitrogen. PKA activity was measured using an ELISA‐based PKA kinase activity assay kit (Abcam) in 3 hearts of each of control, 6‐Bnz and CPT groups. The Table shows PKA activity (ΔOD·μg crude protein−1) of each heart. Figure S4 PVDF membranes containing proteins of the membrane and the cytosol fractions stained with Ponceau‐S for 2 min. The samples of control hearts and hearts treated with 8‐Br (n = 6 in each group) were alternated on the gel and transferred on the membrane. The [file BPH-174-438-s001.pdf]

Supplementary Figure 1

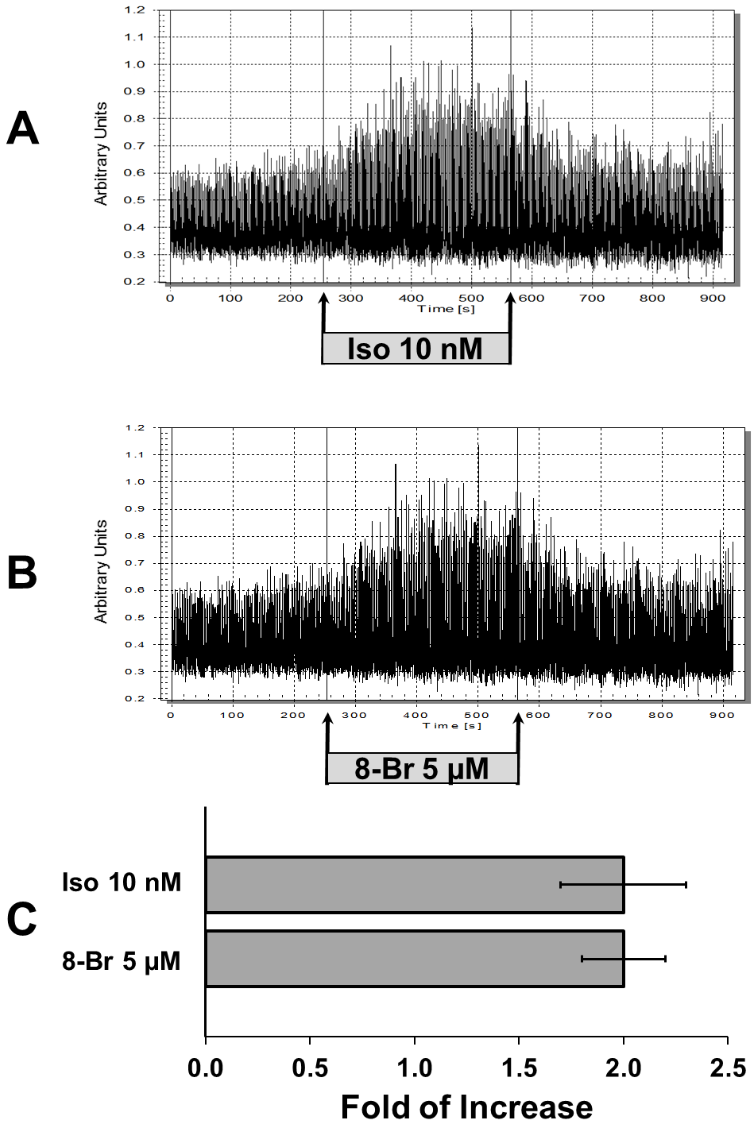

Supplementary Figure 1 – Isoproterenol (10 nM) and 8-Br (5 µM) increased amplitude of Ca^{2+} transients in cardiomyocytes to a similar extent. Groups of cells: Isoproterenol (Iso, n=9); 8-Br (n=7).

Panel A – Representative recording of Ca^{2+} transient in a cardiomyocyte superfused with Iso for 5 min followed by 5 min washout.

Panel B – Representative recording of Ca^{2+} transients in a cardiomyocyte superfused with 8-Br for 5 min followed by 5 min washout.

Panel C – Value of increase in the amplitude of Ca^{2+} transients in cardiomyocytes treated with Iso and 8-Br.

Supplementary Figure 2

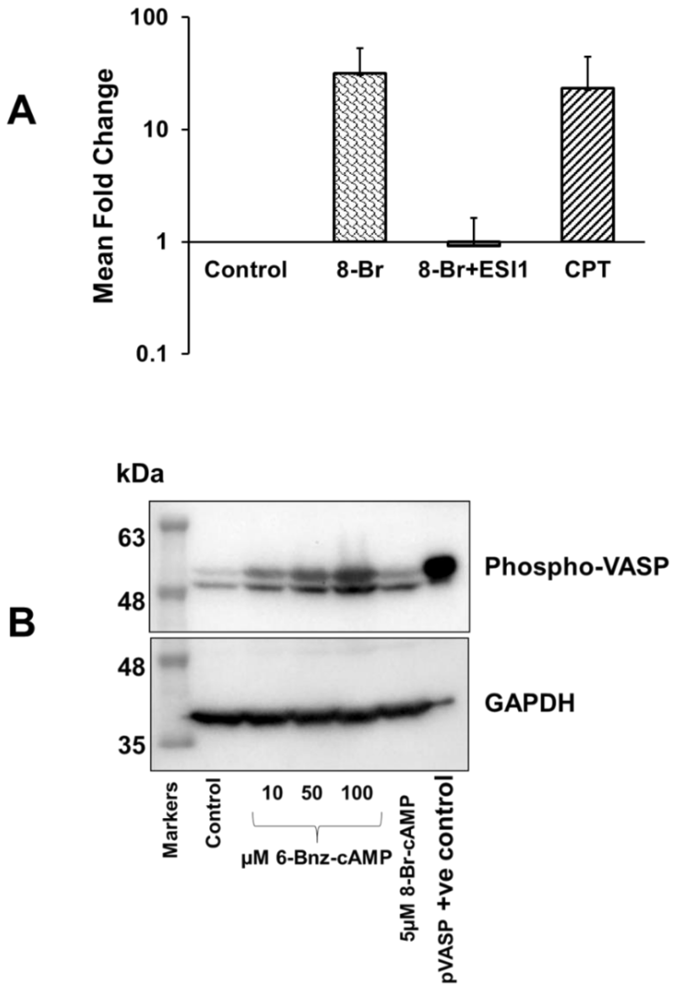

Supplementary Figure 2 – c-fos gene expression in H9C2 cells treated with Epac activators and inhibitor (panel A) and comparison of VASP phosphorylation by 8-Br and 6-Bnz (panel B).

Panel A – c-fos gene expression measured in H9C2 cells incubated at 37ºC for 7 h with either 5 µM 8-Br, 10 µM CPT or 5 µM 8-Br + 1 µM ESI-09. The gene expression was calculated as Fold Change relative to Control. The level of gene expression in Control group was set as 1.

Panel B – a Western blot for phospho-VASP of the lysate of rat heart treated with 8-Br (5 µM) and 6-Bnz (10-100 µM). Control – a lysate of untreated Langendorff-perfused rat heart. pVASP + VE Control – a lysate of rat smooth muscle cells treated with 25 µM Forskolin used as a positive control for PKA activation.

Supplementary Figure 3

Group of Hearts	PKA Activity ΔOD·µg crude protein^{-1}		
	Heart No		
	1	2	3
Control	0.75	0.87	1.07
6-Bnz	3.18	1.57	2.37
CPT	1.48	0.93	0.73

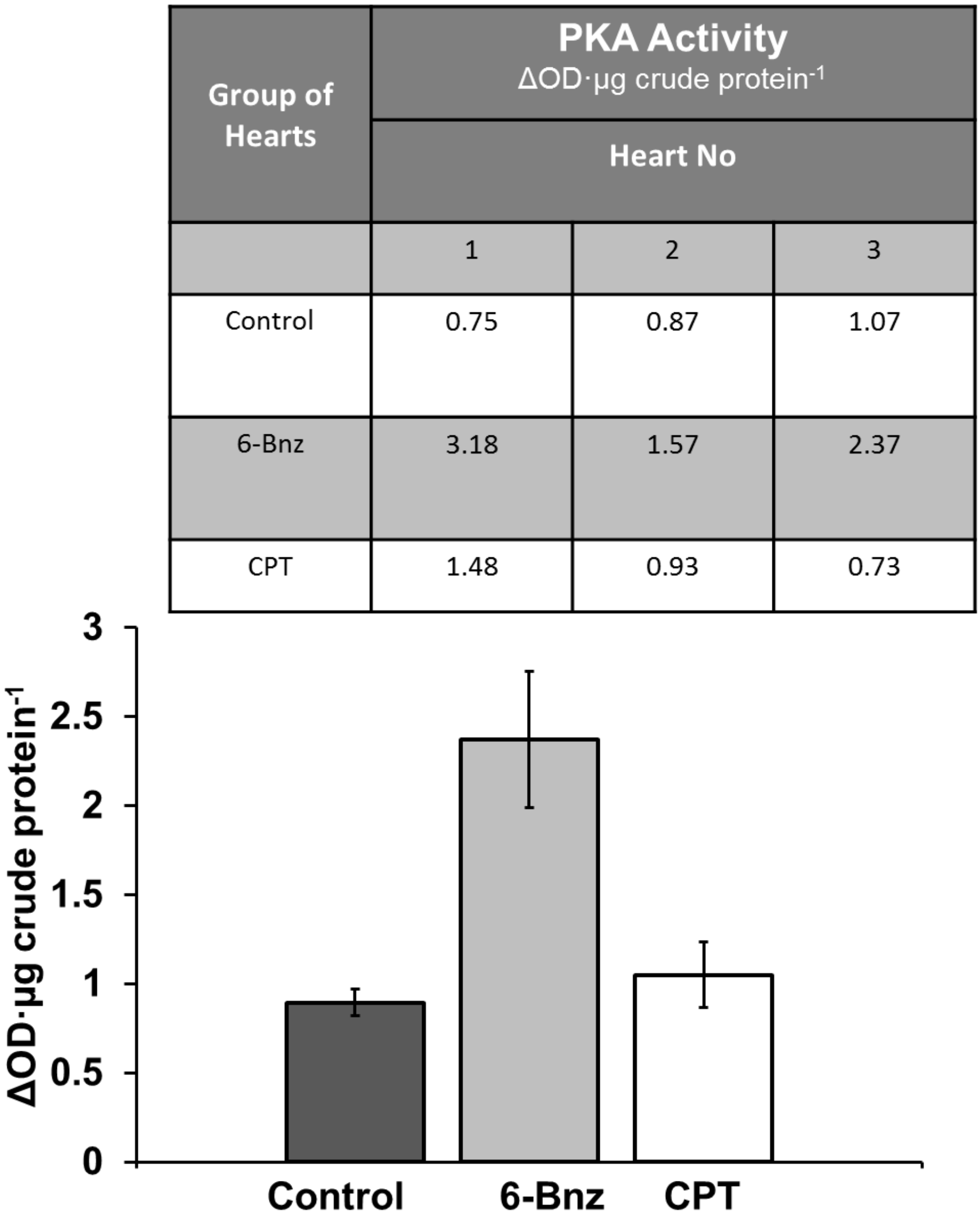

Supplementary Figure 3 – PKA activity in hearts perfused with 10 µM 6-Bnz or 10 µM CPT
The hearts were freeze-clamped at the end of the pre-ischaemic protocol, outlined in the Methods, and powdered under liquid Nitrogen. PKA activity was measured using an ELISA-based PKA kinase activity assay kit (Abcam) in 3 hearts of each of Control, 6-Bnz and CPT groups. The Table shows PKA activity (ΔOD·µg crude protein^{-1}) of each heart.

Supplementary Figure 4

Membrane fraction

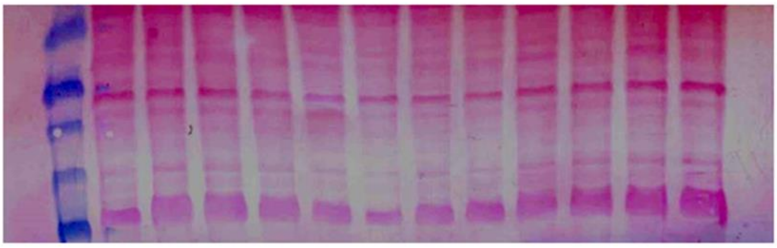

Cytosol fraction

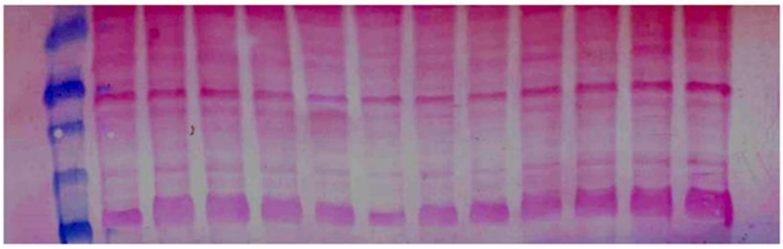

Supplementary Figure 4 – PVDF membranes containing proteins of the membrane and the cytosol fractions stained with Ponceau-S for 2 min. The samples of Control hearts and hearts treated with 8-Br (n = 6 in each group) were alternated on the gel and transferred on the membrane. The figure confirms an even loading of the proteins of different samples.
